# Developing a postgraduate professional education framework for emergency nursing: a co-design approach

**DOI:** 10.1186/s12912-021-00560-z

**Published:** 2021-03-12

**Authors:** Karen A. Theobald, Fiona Maree Coyer, Amanda Jane Henderson, Robyn Fox, Bernadette F. Thomson, Alexandra L. McCarthy

**Affiliations:** 1 School of Nursing, Faculty of Health, Queensland University of Technology, GPO Box 2434, Brisbane, Queensland 4000 Australia; 2 Centre for Health Care Transformation, Q Block, 60 Musk Avenue, Kelvin Grove, Queensland 4059 Australia; 3 Royal Brisbane and Women’s Hospital, Herston, Queensland 4029 Australia; 4 School of Nursing, Midwifery and Social Sciences, Central Queensland University, Brisbane, Queensland 4000 Australia; 5 Princess Alexandra Hospital, 199 Ipswich Road, Woolloongabba, Queensland 4102 Australia; 6 Metro North Hospital and Health Service, Herston, Queensland 4029 Australia; 7 School of Nursing, Social Work and Midwifery, University of Queensland, St Lucia, Queensland 4072 Australia; 8 Mater Health Services, Raymond Terrace, South Brisbane, Queensland 4101 Australia

**Keywords:** Co-design, Postgraduate nursing education, Emergency nursing, Curriculum, Industry-academic, Partnership

## Abstract

**Background:**

Hospital and university service providers invest significant but separate resources into preparing registered nurses to work in the emergency department setting. This results in the duplication of both curricula and resource investment in the health and higher education sectors. This paper describes an evidence-based co-designed study with clinical-academic stakeholders from hospital and university settings.

**Methods:**

The study was informed by evidence-based co-design, using emergency nursing as an exemplar. Eighteen hours of co-design workshops were completed with 21 key clinical-academic stakeholders from hospital and university settings.

**Results:**

Outcomes were matrices synchronising professional and regulatory imperatives of postgraduate nursing coursework; mutually-shaped curriculum content, teaching approaches and assessment strategies relevant for postgraduate education; a new University-Industry Academic Integration Framework; five agreed guiding principles of postgraduate curriculum development for university-industry curriculum co-design; and a Graduate Certificate of Emergency Nursing curriculum exemplar.

**Conclusion:**

Industry-academic service provider co-design can increase the relevance of postgraduate specialist courses in nursing, strengthening the nexus between both entities to advance learning and employability. The study developed strategies and exemplars for future use in any mutually determined academic-industry education partnership.

## Background

In Australia, the health industry and the tertiary sector provide post-registration courses for emergency nurses [1]. These range from practical, competency-based shorter modules to specialty programs to longer courses that are mixed theory and practice, postgraduate degrees. Irrespective of whether a course is delivered by a hospital or a university, education providers have a common goal. That is, to promote students’ higher-level learning, arming them with the knowledge and skills to work from a strong evidence base [2]. To serve this end, the Australian Qualifications Framework (AQF) Council operates as an advisory body to regulate all postgraduate offerings across Australia. Despite said aims, the significant but separate energies invested by industry and universities in postgraduate course design and delivery often results in the unnecessary duplication of content and resources [3]. Notwithstanding the global demand for postgraduate courses, evidenced for example by 1700 United States universities offering Masters-level programs [4] and many institutions in the United Kingdom offering Graduate Certificate and Doctoral Preparation courses [5], there are critical gaps in postgraduate offerings [3] in Australia. Courses offered by higher education providers (HEP) are based on industry need and are developed and delivered with significant industry input [6]. For example, in the Australian context postgraduate courses are overseen by course advisory committees with membership comprising of academics, key industry and professional organisation stakeholders [7]. However, these advisory committees are often implemented ‘after the fact’ – when university stakeholders have already undertaken most of the course design and often delivered it.

This study was a collaboration of academics and educators, clinicians, postgraduate emergency nursing students, administrators and nursing regulators. The co-designed partnership offered a new way to design learning based on a culture of shared decisions, relevance and agility, created and agreed from the outset. This enabled a range of stakeholders to contribute and have their needs heard [8, 9]. Other approaches, such as a Delphi method, would not have given us the rich engagement and ongoing feedback that we were able to achieve through the lens of the co-design approach [8].

## Methods

### Aim

Our aim was to be mutually involved from the beginning of course conceptualisation and design. Collectively we sought to better harness current collaborations through the development of an industry-academic postgraduate education framework that integrated the goals of both the health industry and higher education service sectors. This co-design process would ensure course credibility and relevance while maximising postgraduate students’ professional learning. Importantly, inviting students, administrators and nurse regulators to work together in co-design, added new perspectives to the process.

### Design

This evidence-based co-designed study involved three workshops (18 h in total) offered in June and December 2016 and May 2017. These workshops were supplemented with iterative between-workshop email and personal consultation with participants. The study was informed by the evidence-based co-design approach in which collaborative collective creativity, which recognises diverse expertise, drives the development of purposeful enterprise that seeks to optimise end-user satisfaction and enhance product and process outcomes [8]. Exemplified by Ogrin et al. [9], the human-centric collaborative approach aims to bring diverse stakeholder skills and contextual knowledge to product (in this case, curriculum) development and delivery through the development of knowledge and generation of mutually-agreed principles [8]. Participants, including stakeholders in leadership capacities as well as product and process consumers, generate insight and are supported by key designers, researchers and leaders in establishing partnerships to effect sustainable educational change.

### Participants

Purposive sampling ensured key participants were included. A total of 38 stakeholders consented with up to 55% actively participating, representing a range of invested party perspectives at each workshop. These were the Chair, National College of Emergency Nursing Australasia (CENA) Credentialing Committee, CENA Queensland President and other representatives of CENA; university-based curriculum experts, emergency nursing course coordinators and past and present postgraduate curriculum directors from the two partner universities; past and present postgraduate students of the two partner universities; emergency nursing educators and clinicians from six metropolitan hospitals in Brisbane; the Executive Directors of Nursing from Metro South and North, Directors of Nursing and Directors of Nursing Education from the partner health services; and one credentialing expert from the Office of the Chief Nursing and Midwifery Officer Queensland. Nursing experience ranged from 12 months post initial registered nurse qualification, to 45 years. Participants’ professional qualifications ranged from a Bachelor degree to Doctor of Philosophy.

### Setting

The study was conducted at two health services and two university settings. These settings represent two major metropolitan universities providing postgraduate nursing education courses with student enrolments across Australia and two major health services in South East Queensland providing quaternary-level emergency health care services. Emergency nursing was selected to be the focus of the study as emergency postgraduate courses were well enrolled in both university partners. Further, these settings were chosen for the existing close collaborative relationships and partners who showed the initiative to take postgraduate curriculum design to a new level.

### Data collection

This study used co-design method, therefore a range of strategies during and after the workshops were used to generate data. The workshops were part of an iterative process with Workshop 1 informing Workshop 2, which in turn informed Workshop 3.

#### Within the workshops

The workshops methodically explored topics including enablers and barriers to industry-academic mutual development and delivery of postgraduate curricula; the conditions of postgraduate industry–academic engagement; compulsory content of postgraduate emergency nursing curricula across settings; and relevant teaching, learning, assessment and quality assurance approaches. Proactive partnerships were promoted in the workshops by focusing on the participants’ common goals. We aimed for reciprocal and co-operative communication processes. In each workshop we used these strategies to establish a common identity as an educational ‘community of practice’ for postgraduate emergency nursing students — a community that aimed to negotiate a pathway for the joint development and potential delivery of a postgraduate emergency nursing course.

Consistent with evidence-based co-design approaches we explored the interface between students, regulatory bodies, universities and health services that customarily have different (but not mutually exclusive) educational goals and tend to operate under different (but not mutually exclusive) norms and performance indicators. Each workshop was led by one Chief Investigator; other Chief Investigators were embedded with participants to organise and summarise group outputs.

**Fig. 1:**
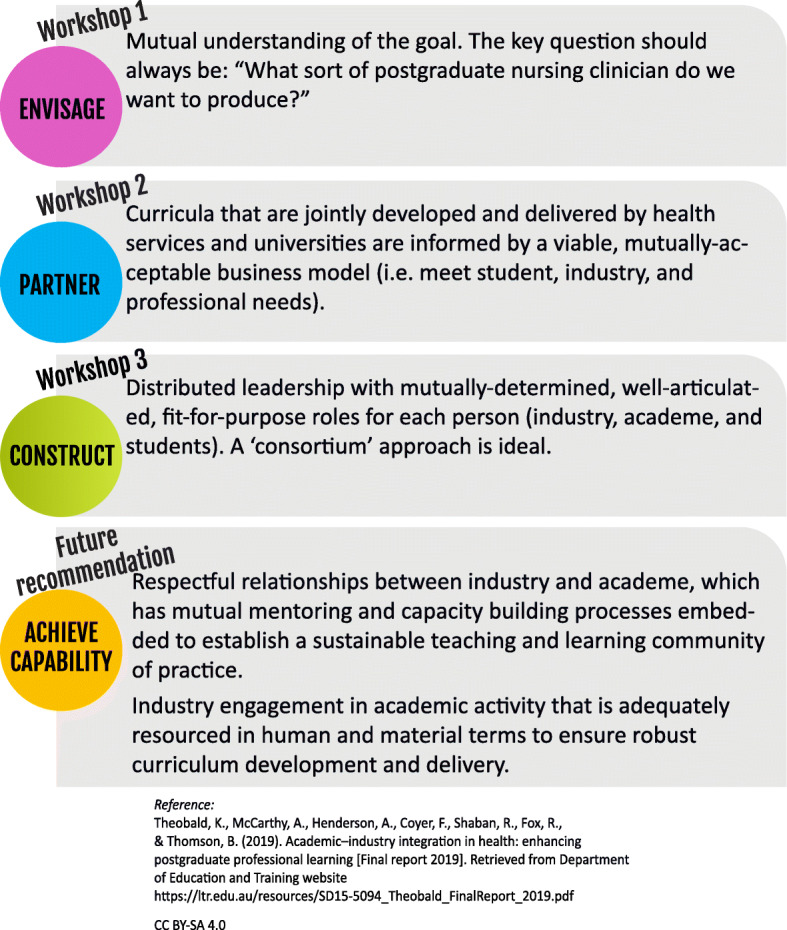
Guiding principles for postgraduate curriculum transformation

**Fig. 2:**
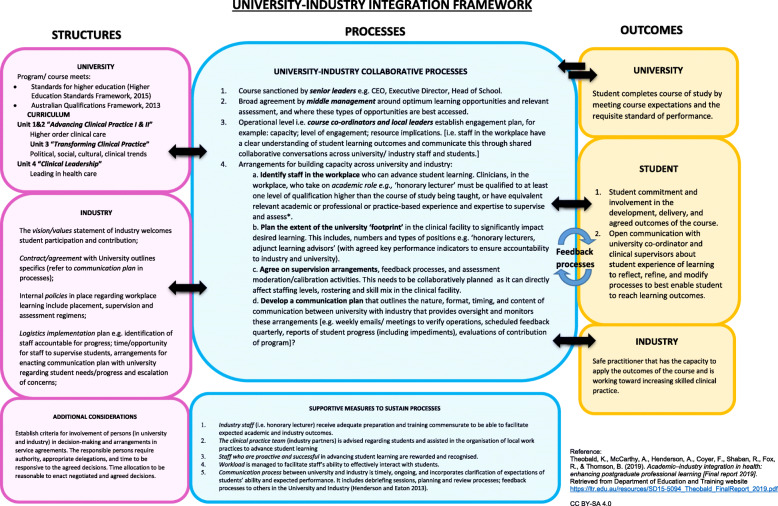
University-industry integration framework

The first workshop, exemplified by Step 1 of the guiding principles for postgraduate curriculum transformation model (Fig. 1), established the agreed evidence for development of the University-Industry Integration Framework (Fig. 2) and envisaged a mutual understanding of the conceptual goal: “What sort of postgraduate nursing clinician do we want to produce?” In workshop two (December 2016), matrices (Tables 1 and 2) were mutually developed that described synchronisation of professional and regulatory imperatives. This included partnerships and collaborative enterprise across the health and university service sectors informed by a feasible business model that met student, industry and professional needs. In the final workshop (May 2017), the Framework (Fig. 2), guided by distributed leadership with mutually-determined, fully-articulated, fit-for-purpose roles for each stakeholder, was finalised.

**Table 1:** Matrix 1: harmonisation of professional and regulatory imperatives for postgraduate coursework nursing education

Level of nursing	AQF level	National nurse standard or reference	Curriculum content	Curriculum nature
Registered nurse	7	NMBA Registered Nurse Standards for Practice 2016 [10]	NMBA-legislated educational requirements	Competency-based as per NMBA standards
Domain-specific nurse	8	NMBA Registered Nurse Standards for Practice 2016 [10]	• Specialty college or association standards (where these exist) • Actual and emergent imperatives in specialty health care delivery articulated by industry reference groups	Competency-based as per NMBA Standards
Advanced practice nurse	9	NMBA Fact Sheet on Advanced Practice Nursing 2019 [11]; and Identifying advanced practice: A national survey of a nursing workforce Gardner et al., 2016 [12]	• Will vary slightly according to whether nurse is classified as ‘clinical’, ‘consultative’ or ‘classical’ [12] advanced practice nurse • Actual and emergent imperatives in health care delivery articulated by industry reference groups	Theory-based (no NMBA standards exist)
Nurse practitioner	9	NMBA Nurse Practitioner Standards for Practice 2014 [13]	NMBA-legislated educational requirements	Theory- and competency-based as per NMBA standards

Reference: Theobald, K., McCarthy, A., Henderson, A., Coyer, F., Shaban, R., Fox, R., & Thomson, B. (2019). *Academic–industry integration in health: enhancing postgraduate professional learning [Final report 2019]*. Retrieved from Department of Education and Training website https://ltr.edu.au/resources/SD15-5094_Theobald_FinalReport_2019.pdf. CC BY-SA 4.0 https://creativecommons.org/licenses/by-sa/4.0/

**Table 2:** Matrix 2: mutual expectations to ensure teaching and learning quality and employability, graduates in postgraduate nursing coursework

**Guiding principles**	Curricula are underpinned by: • Mutual understanding of the goal postgraduate attributes. The key question should always be: “What sort of postgraduate do we want to produce?” • Clear articulation of mutual curriculum values via a conceptual model of nursing practice, such as the Strong Model • Equal industry and academic referencing • Respectful relationships between industry and academe, which has mutual mentoring and capacity building processes embedded to establish a sustainable teaching and learning community of practice • Distributed leadership with mutually-determined, well-articulated, fit-for-purpose roles for each person. A ‘consortium’ approach is ideal. • Industry engagement in academic activity that is adequately resourced in human and material terms to ensure robust curriculum development and delivery • Mutual articulation and understanding of student, organisational and professional needs			• Mutual understanding of entry and exit requirements • Robust evidence • Explicit relationships between, and integration of, all course content and strategies • Viable inter-institutional methods of assuring learning and teaching standards • Evaluation underpinned by mutually-relevant metrics • Curricula that are jointly developed and delivered by health services and universities are informed by a viable, mutually-acceptable business model • Accountability for implementing agreed actions is built in • Articulation process between health service and university courses should be flexible and seamless for students, with clearly articulated processes.			
**Course development**	• Agreed terms of industry-academic engagement established; e.g.: ◦ Who will be involved in the clinical learning agenda (students, industry representatives, academics, consumers), and how will this be determined ◦ Extent and mode of their involvement ◦ Methods to identify and resolve clinical, academic and regulatory issues determined ◦ Articulation and actioning of enablers to engagement ◦ Articulation of and solution to barriers to engagement ◦ Timing and format of joint communications ◦ How distributed leadership will be enacted			• Forward mapping: Developmental learning outcomes that take student from the known to the unknown (i.e. equivalent to knowledge→skills→application; or competence→capability) are scaffolded throughout the course, units and assessments • Back mapping: The ideal course graduate is the starting point. Backward design begins with the objectives of the course—what students are expected to learn and be able to do on graduation; it then proceeds “backward” to create learning experiences and strategies to achieve those goals • Openness to a ‘pick and mix’ or ‘shopping cart’ approach; that is, modular approach where all modules are focused on learning outcomes, but the mix of modules is adaptable to student needs			
**Course delivery**	**Course outcomes**	**Content**	**Contexts of learning**	**Teaching approaches**	**Learning approaches**	**Assessment principles**	**Assessment types**
	Target postgraduate capabilities as per Strong Model are articulated and woven through all aspects of course	Specialty college or association-specified content that is competency-driven (AQF Level 8) Driven by discrete context of practice and explicitly underpinned by theory as well as competency (AQF Level 9) Build on resources health services and universities have already developed; e.g. advanced life support in university course assumes achievement of basic life support competency in hospital	Acute settings Community settings Policy settings Simulation lab Face-to-face (lectures, tutorials) Online (synchronous and asynchronous)	Scaffolded from information transmission, to concept acquisition (knowledge), to concept development (skills), to concept change (application) All teaching strategies and assessment processes scaffold developmental learning outcomes Interdisciplinary teaching Cross-specialisation teaching (e.g. emergency nurses teach physical assessment)	Industry case study Clinical simulation Industry mentoring Industry placement Rotational placements Developing patient plans Self-directed learning Group learning Individual learning Remote simulation	Clinical competency assessments and processes are consistent with the NMBA position statement on assessing standards for registered nursing practice (2015); e.g., clinical competency assessments are performance-based and undertaken in the practice context by assessors who are appropriately clinically and academically prepared. Clinical competencies should be assessed by clinicians, theoretical assessments by academics but all jointly decided on, depending on context. Flexibility in clinical competency assessment fundamental; e.g. viva, observation, simulation, performed via telehealth. Depends on what is available in context Academic assessments and processes are congruent with Australian Tertiary Education Quality Standards Agency (TEQSA) imperatives Professional curriculum development and delivery opportunities enabled for teachers, especially industry-based teachers Standard processes implemented to ensure a) accurate calibration of markers and b) robust and transparent moderation processes across academic and industry contexts Mutual agreement on performance expectations e.g. what is a pass? Dilemma-based (i.e. problem-based) learning grounded in real practice problems heighten engagement and reduce incidence of plagiarism	Competency assessment Practice audit Create policy and practice guideline Present in-service Classical essay Classical multichoice and short answer exams Portfolios of key competencies and capabilities Small or large thesis or project
**Course evaluation**	Processes established for: • Objective student evaluation • Regular and systematic inter-institutional quality assurance (benchmarking), review and moderation of curricula						

Reference: Theobald, K., McCarthy, A., Henderson, A., Coyer, F., Shaban, R., Fox, R., & Thomson, B. (2019). *Academic–industry integration in health: enhancing postgraduate professional learning [Final report 2019]*. Retrieved from Department of Education and Training website https://ltr.edu.au/resources/SD15-5094_Theobald_FinalReport_2019.pdf. CC BY-SA 4.0 https://creativecommons.org/licenses/by-sa/4.0/

Data collection activities included evidence-based co-design techniques such as ice-breaking sessions, small and large group brainstorming, group generation of themes using post-it notes and buzz groups. With the agreement of participants, written outputs of group work, such as mind maps, were retained and intensive field noting of verbal outputs based on informal interviews was undertaken by a Chief Investigator during each workshop. Other research team members made field notes while facilitating break-out groups and also provided group-approved summaries of key points, which were included in the analysis. Due to the highly interactive nature of workshops and number of participants, it was not useful to audio-record the proceedings.

#### Post-workshop

Post-workshop emails were sent to all participants requesting feedback and input on workshop summaries, materials and outcomes, including questions with reference to the literature. Five current students provided feedback on materials that were developed from the three workshops between May and June 2017. Past students also contributed to Workshops 1 and 3. After the workshops, two focus groups (50 & 60 min each) were conducted with five students from two different universities, who were enrolled as internal and external students. Two students met with one researcher individually for a 35 and 45-min interview. Comprehensive field notes were made by the Chief Investigator during focus groups and interviews. Feedback from students provided insight of role in curricula development, implementation and evaluation, as well as applicability of the graduate certificate example and university-industry framework.

### Data analysis

The workshops, follow-up consultations, focus groups and interviews generated qualitative data. Analysis of these data was iterative, moving from initial codes to researcher-created ideas and the formation of themes. The analysis moved from descriptive to abstract analytical concepts and culminated in two main themes [14, 15]. The process was led by two experienced qualitative researchers and involved ongoing dialogue with all researchers with an awareness of testing validity through the interpretations generated [14].

## Results

The number of participants able to attend all or part of each workshop was influenced by clinical demands at the time and ranged from nine (Workshop 3), to 16 (Workshop 2) and 21 (Workshop 1).

The data formed eight categories that fell within two main themes. These two themes are summarised in the two matrices presented in Tables 1 and 2. Matrix 1 [or theme 1] (Table 1) harmonises the first two categories: the mandatory factors that shape postgraduate nursing curricula in Australia and the need for a unifying model of nursing practice across educational contexts. Matrix 2 [theme 2] (Table 2) systematically articulates the remaining six categories. These cover the principles and processes that should be thought-out when developing and delivering emergency nursing curricula. These include good practice principles of professional learning; types of professional learning; stakeholder terms of engagement; scaffolding of teaching and learning; harnessing the diverse contexts of learning; and assuring course quality. The data were also drawn on to develop an interim framework (Fig. 2). The categorised data were also provided to participants for review and feedback.

The two matrices aligned the postgraduate emergency nursing education goals and curriculum development processes of the local health care service industry, national health care regulators and national higher education bodies, as well as the professional learning and credentialing needs of students and their employers. A robust thread running through both themes was the importance of work-integrated learning to prepare postgraduate students for practice. The culmination was mutual agreement of an operational framework to optimise learning of emergency nursing based on an academic-industry-student collaborative approach (Fig. 2) as well as the creation of a graduate certificate in emergency nursing curriculum exemplar. This led to establishment of principles to guide future curriculum development, implementation and evaluation.

Five guiding principles (Fig. 1) of curriculum development, implementation and evaluation were then established to inform the processes subsequently used in the study. Beneficial in the establishment, maintenance and development of a co-curricular distributed leadership partnership, the principles demarcate and represent the evolutionary steps involved in moving through design and creation phases to achieving capability with tangible outcomes and future direction. Emerging from curriculum development milestones and processes undertaken during the workshop series, the principles articulate the architecture of collaborative, linear and iterative scaffolded processes through which participants can transition while designing and constructing sustainable and suitable curricula. As such, a key outcome of Workshop 3 was the finalisation of a clinical-academic integration strategy and framework construction. This resulted out of a consortium approach of distributed leadership and jointly-determined, fully-articulated, fit-for-purpose roles for industry, university and student roles – the third principle.

However, to achieve capability, it was determined that respectful relationships between industry and academe must be maintained, and that affiliations that embedded joint mentoring and capacity building processes must be established. This would ensure a viable longer-term teaching and learning community of practice. To ensure robust development and delivery, successful postgraduate curriculum transformation also relies on health service provider engagement in appropriately-resourced academic activity. These were the final two guiding principles, delineated with the intention of providing future recommendations. The adoption of five guiding principles potentially supports the sustainable implementation of co-curricular partnerships.

Matrix 2 (Table 2) captures expectations of the various stakeholders with respect to learning and teaching quality and enhancing student employability. The participants considered it essential that all stakeholders understand and articulate the good practice principles of nursing curriculum development and delivery before they determine teaching and learning processes. Participant summaries generated through workshops are represented in Table 2 and Figs. 1 and 2.

Process issues were also important for participants, as summarised in Matrix 2. They encouraged stakeholders to formulate agreed terms of engagement from the outset. For example, explicit discussions occurred about who should be involved in the professional learning agenda, how to identify and resolve health service, university, credentialing and regulatory issues, articulation of enablers, barriers and solutions to engagement, the most desirable timing and format of joint communications, and how best to enact distributed leadership taking place *before* course development. It was also considered important to tease out technical issues, such as how best to scaffold learning. Participants further identified that workable cross-institutional ways of ensuring learning and teaching standards and undertaking mutual peer review and moderation were essential to ensure courses met student needs. While resolution of these issues was beyond the scope of this study, these notions certainly warrant further research as to how they might be achieved.

### Graduate certificate of emergency nursing curriculum exemplar

In harmonisation with the principles and objectives that transpired through creation of a framework, an exemplar Graduate Certificate of Emergency Nursing was developed. Key facets were also delineated as part of the second matrix. It is positioned as a course outline that can be used by any health service or university, an instrument that guides the development of learning and teaching strategies and curriculum content [14].

### A fit-for-purpose university-industry integration framework

An important issue identified during workshops was the need to harmonise regulatory and other imperatives, while ensuring that course content and level of learning are congruent with the student’s target level of nursing practice. In essence, the course should overtly and systematically prepare the nurse for either specialty emergency practice, advanced emergency nursing practice or for a nurse practitioner position. Participants further identified that postgraduate education is often undertaken to enhance employability. Hence, successfully-completed courses should readily help students to progress through the levels of practice embodied in the nursing career structure. Workshop consensus was that to ensure harmonisation and to assist career progression, a unifying model of nursing practice that accurately reflects the target practice profile and maps to the career pathway is needed. This would underpin all postgraduate courses, irrespective of whether they are offered by universities or health services. Subsequently, a university-industry integration framework (Fig. 2) was developed, to represent the structures, processes and outcomes required in developing and delivering postgraduate nursing courses that are co-created in university-industry service provider partnerships.

## Discussion

The most important finding arising from this study was the overwhelming desire for industry to be involved in all aspects of university postgraduate emergency course development and for universities to likewise be involved in courses developed and delivered by industry (Fig. 2). Traditionally stakeholder involvement in course advisory committees provides a controlled environment for engagement, yet in this project, co-design enabled a more equal playing field of all stakeholder contributions. The emphasis all participants placed on mutual involvement was significant, but deliberations indicated that currently, it is not well-operationalised. This is despite the fact that tertiary education in Australia is regulated by the Tertiary Education Quality Standards Agency (TEQSA) [16] stipulate that universities engage regularly with course advisory committees comprising industry stakeholders to ensure the relevance and currency of their curricula. Similarly, health services often seek university advice to ensure the congruence of their professional development offerings with AQF Level 8 courses to provide a postgraduate articulation pathway for their emergency nursing students. Graduate Transition Programs offered in Queensland, Australia are good examples. These hospital-based programs deliver an entry to specialty program to newly-graduated/employed registered nurses, which can often be credited towards university study [7]. However, discussions during workshops clearly indicated that while both universities and hospitals do seek input from the other, this tends to occur when courses are already conceptualised and largely developed. In contrast, Henderson and Creedy [17] argue that the best student learning experiences occur when clinical content, context and teaching approaches are negotiated from the beginning of course development.

Consistent with the literature [18–21] the data indicated that it was important to have a range of professional learning experiences and related assessment methodologies (e.g., industry case studies, simulation, industry mentoring, industry placements) within curricula and adapted to context. Work-integrated learning and assessment for a rural emergency nurse, for example, might be made most relevant if metropolitan placements, or video-conferenced simulations, are embedded. As presented in Matrix 1, the educational value of learning-in-place warrants explicit acknowledgment within university postgraduate nursing curricula [12]; however, workplaces should also understand both what students *bring* to work-integrated learning placements and what they *need* from the workplace to maximise their learning [22].

The risk of universities providing sub-standard learning experiences for nurses without deep industry engagement was also highlighted in this study. This is corroborated by Australian research in the higher degree context, which clearly demonstrated the ill-preparedness of graduate and postgraduate students for the ‘world of work’ [20, 23]. Substantial evidence from multiple practice disciplines further indicates that deep learning can only occur if industry and disciplinary bodies are explicitly engaged in university course development and delivery [6]. Mutually determined work-integrated learning approaches appear to be the best way to enable authentic learning environments [6, 20, 24]. Hence, Matrix 2 is anchored in principles such as ensuring industry referencing throughout course development and delivery; initiating and sustaining collegial relationships; properly resourcing industry involvement in curricula; fully understanding the students’ learning needs by involving them in course development; and ensuring that curriculum currency is maintained.

The five guiding principles created from this study are starting points for those looking to co-design and implement a postgraduate curriculum in partnership. Our experiences of industry and academe working respectfully and collectively culminated in shared outcomes grounded in these principles. Further, the value that all participants placed on integrating workplace-based learning into university courses targeting domain-specific and advanced practice nurses [11], as well as nurse practitioners [13], warrants consideration. Sociocultural theories of workplace learning propose that optimal learning outcomes are the result of students’ active participation in clinical activities during their study, coupled with their ‘real-life’ learning interactions with the complex and dynamic clinical work environment [18, 22, 25]. Hence, alignment of the industry-academic agenda through well-designed work-integrated learning and assessment experiences increases the relevance of postgraduate courses in emergency nursing (Table 2).

The co-designed Graduate Certificate of Emergency Nursing exemplar developed during this study encompasses the requirements of industry and aims to provide students with quality learning and useable course outcomes. It embraces the imperatives and mutual goals of both industry and universities, while harmonising diverse pedagogical approaches. Devised as a means of strengthening industry-university collaboration, it serves to bridge the current gap between practical capabilities and theoretical scaffolding of learning (reflective of AQF compliance), while meeting the regulatory requirements of the Nursing and Midwifery Board of Australia [10, 11].

## Implications

This study has highlighted the potential of an industry-academic agenda increasing the relevance of postgraduate specialist courses in health, reinforcing the connection between industry and higher education to promote postgraduate learning and employability. Figure 2 provides a unique future blueprint for employability skills to build more collaborative partnerships between employers, industry, higher education and professional bodies in health. In the future, we hope to test the Framework formally, along with its emergency nursing exemplar. The aim is to ensure the transparency of the Framework, promote sustainability of the embedded principles, develop and operationalise joint governance processes and support continuing development of such educational partnerships.

## Limitations of the study

We acknowledge the lack of generalisability of this study’s findings, namely that the findings are context-specific to health services and universities involved. However, given the depth of participants’ background, from clinical care to professional organisation and academic representatives, we suggest that our framework is considered, it can be adapted to other postgraduate nursing contexts.

## Conclusions

This study presented a joint exploration of strategies to design nursing curricula, with the objective of producing work-ready postgraduates who benefit from a thoughtful blend of clinical and university learning experiences. We also illuminated the role of local hospitals and universities in preparing registered nurses for specialist employment and explored how to embed these ideas within curricula using the exemplar of postgraduate emergency nursing. Consensus from the workshops indicates that in the ideal scenario, the framework could be used widely by local health services and universities to guide their joint development of courses, where work-integrated university-level teaching and learning are embedded. The strategies and exemplars developed in this study offer guidance for any professional education provider. The framework exemplifies a spirit of consultation and collaboration, valuing student voice that could usefully guide future joint curriculum development and delivery in other postgraduate nursing specialities.

## Data Availability

The datasets used and/or analysed during the current study are available from the corresponding author on reasonable request.

## Abbreviations

AQF: Australian Qualifications Framework
HEP: Higher education providers
CENA: College of Emergency Nursing Australasia
TEQSA: Tertiary Education Quality Standards Agency
